# Supplementary material for: Comparison of temperature and doping dependence of elastoresistivity near a putative nematic quantum critical point
Source: Nat Commun. 2022 Feb 23;13:1011. doi: 10.1038/s41467-022-28583-3 (PMC8866430; doi:10.1038/s41467-022-28583-3)
Supplement: Supplementary file 1 — Supplementary Information [file 41467_2022_28583_MOESM1_ESM.pdf]

Supplementary information for Comparison of temperature and doping dependence of elastoresistivity near a putative nematic quantum critical point

J. C. Palmstrom,[1,2,3] P. Walmsley,[1,2,3] J. A. W. Straquadine,[1,2,3] M. E. Sorensen,[1,4,3] S. T. Hannahs,[5] D. H. Burns,[6] and I. R. Fisher[1,2,3]

[1]*Geballe Laboratory for Advanced Materials, Stanford University, Stanford, CA 94305, USA*
[2]*Department of Applied Physics, Stanford University, Stanford, CA 94305, USA*
[3]*Stanford Institute for Materials and Energy Sciences, SLAC National Accelerator Laboratory, Menlo Park, CA 94025, USA*
[4]*Department of Physics, Stanford University, Stanford, CA 94305, USA*
[5]*National High Magnetic Field Laboratory, Florida State University, Tallahassee, FL 32310, USA*
[6]*Department of Geological Sciences, Stanford University, Stanford, CA 94305, USA*

(Dated: January 3, 2022)

A. Supplementary Note 1: Experimental Errors

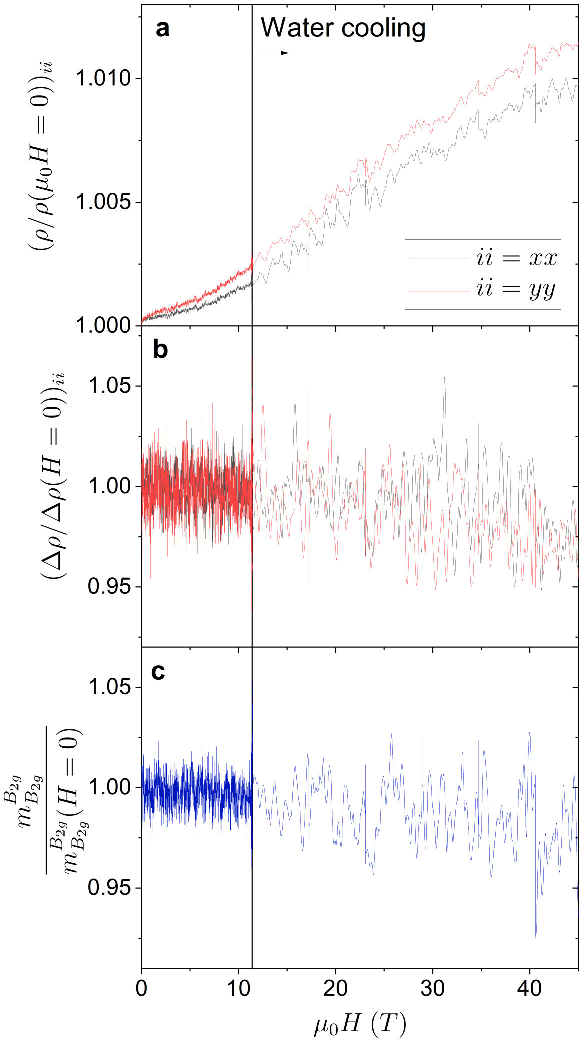

Supplementary Figure 1. **A representative raw data trace as a function of field from 0 T to 45 T.** Data are taken on sample x=0.0793 at 33 K and no Savitzky-Golay filter has been applied. Fields above 11.4 T (vertical black line) require water cooling of the magnet which introduces additional background noise.

Strain was measured with a bi-directional resistive strain gauge from Micro-Measurements (Part. No. WK-06-062TT-350). The strain gauges were measured in a Wheatstone bridge configuration with the three balance resistors at room temperature. Since strain was simultaneously measured along two orthogonal directions we were able to correct for the transverse strain sensitivity of each strain gauge using the manufacturer's provided calibration. We also accounted for the temperature dependence of the gauge factor. The largest uncertainties in the measurement of the oscillating strain experienced by the strain gauge are from balancing the bridge with the assumption that the line resistance of the cryostat was the thermalized value at either 30 K or 300 K depending on the temperature the bridge was balanced. Other smaller sources of error ($< 0.6\%$) include the thermal output of the strain gauge, the magnetoresistance of the gauge, and uncertainty in the manufacturer's provided specifications. Overall error is estimated to be between 3%-5%, with a value of 4.1% used for the calculations in this manuscript. The measurement noise was quantified by taking a rolling standard deviation over a 1 K window (4 K for the two most overdoped samples). Additionally two samples, $x = 0.0722$ and $x = 0.096$, exhibited sharp shifts in the apparent measured value of $m_{B_{2g}}^{B_{2g}}$ on the order of 10% which we tentatively attribute to changes in the current path through the silver paint contacts due to mechanical shifts during cooling. These three sources of error are included in the y-error bars in Figure 3.

Pre-Savitzky-Golay filtering resistivity and elastoresistivity data as a function of field are shown in Supplementary Figure 1. While there is a finite magnetoresistance in the resistivity response, there is no contribution from the Hall effect since these are longitudinal resistivity measurements. The elastoresistivity– i.e. the derivative of the resistivity with respect to strain, has no discernible field dependence. Data from 0 T-11.4 T are taken during the initial field ramp of the outsert superconducting magnet. Magnetic fields above 11.4 T are reached with the resistive insert magnet which requires water cooling and introduces substantial noise, presumably due to vibrations.

B. Supplementary Note 2: Extracting $\mathbf{x}_c$

In the Fe-based superconductors there is an electronically driven tetragonal-to-orthorhombic phase transition. There are two critical temperatures to consider for this phase transition, the 'bare' electronic ordering temperature (Θ), this is the temperature at which the system would order if there were no coupling to the lattice, and the actual ordering temperature of the system (T_s). Even though this is an electronically driven phase transition, the presence of the lattice and the cooperative effect of a finite electron-lattice coupling means that the critical temperature for the electron-lattice system will occur at a higher temperature than the bare electronic ordering temperature (i.e. $T_s \geq \Theta$).

By measuring the strain experienced by the material, elastoresistivity measurements probe the 'bare' electronic nematic fluctuations of the system. Meaning, the inferred nematic susceptibility is purely electronic in origin and independent of any cooperative effects from lattice softening. Therefore the measured elastoresistivity response should diverge at the 'bare' electronic nematic quantum critical point (x_c^{bare}), if such a point exists in the system.

In practice, for the Co-doped $BaFe_2As_2$ system, x_c^{bare} is difficult to determine. The electronic nematic susceptibility, as measured by the elastoresistivity response, does not follow a known form as a function of temperature close to the putative critical point and measurement of the elastoresistivity through the 'bare' electronic transition is precluded due to the presence of the higher temperature structural transition for underdoped compositions. In the isovalently substituted $BaFe_2(As_{1-x}P_x)_2$, the elastoresistivity follows a Curie-Weiss functional form for significantly higher dopings, including near the putative nematic quantum critical point where the 'bare' electronic nematic transition temperature goes through zero[1]. This point was found to correspond to between $x = 0.032$ and $x = 0.036$, i.e. near optimal doping and close to where the structural transition is suppressed to zero. Therefore, for the analysis presented in this manuscript we approximate x_c^{bare} as x_c.

To extract x_c, we performed four point resistivity measurements on free standing bar samples for $x = 0.0606$, 0.0616, 0.0631, and 0.0648 (Supplementary Figure 2). For far underdoped compositions ($x < 0.051$) the structural transition can be identified by a mean-field like step in the temperature derivative of the resistivity ($-\frac{d(\rho/\rho(T=300\ K))}{dT}$). In zero field this feature broadens with doping and disappears above $x = 0.051$[2]. With the suppression of superconductivity in 45 T magnetic fields, signatures of the structural transition reappear in resistivity measurements. The features are still extremely broad and merge with the signature of the antiferromagnetic transition. From the temperature derivative of resistivity alone (Supplementary Figure 2) it is not possible to precisely determine the structural transition temperature. The onset of the structural transition can be bounded by the sharp change in the slope of $-\frac{d(\rho/\rho(T=300\ K))}{dT}$. Since there is no separation between the broad mean-field step associated with the structural transition and the onset of the antiferromagnetic order, the lower bound is set by the downturn of $-\frac{d(\rho/\rho(T=300\ K))}{dT}$. This downturn for far underdoped compositions with sharp transitions occurs at temperatures below the subsequent antiferromagnetic transition. This is separate from the sharp downturn induced by the onset of superconductivity. For $x = 0.0648$ there is no resolvable down turn associated with the antiferromagnetic transition before the onset of

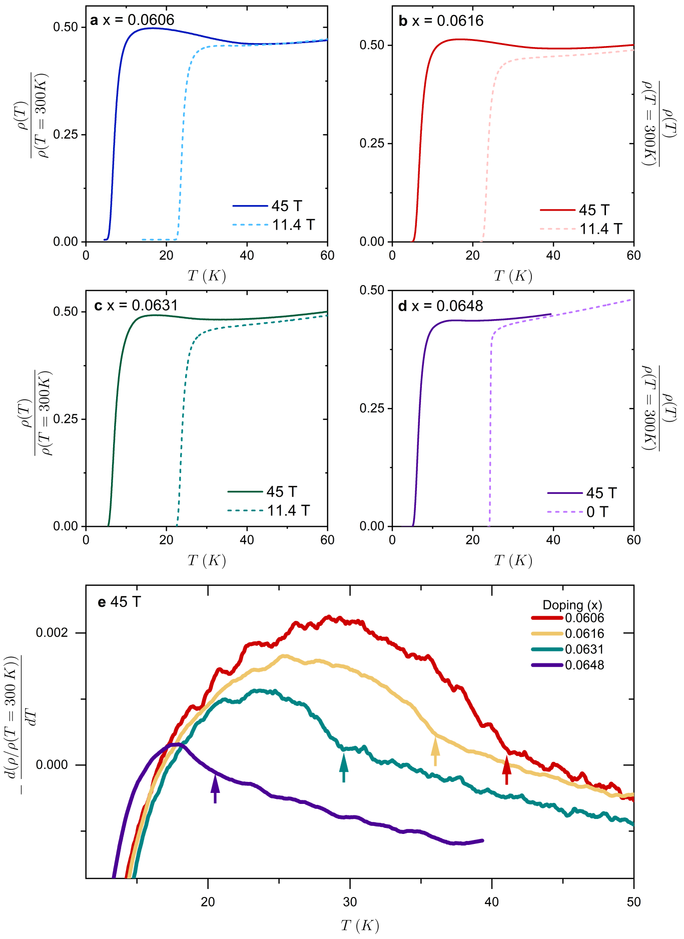

Supplementary Figure 2. **The field and temperature dependence of the resistivity for free standing samples with** x = 0.0606**, 0.0616, 0.0631, and 0.0648**. **a-d** Comparison of the high and low magnetic field resistivity vs temperature response. The data are normalized by the zero field room temperature values. **e** The temperature derivative of the high field resistivity response. Vertical arrows mark the onset of a broad step in $-\frac{d(\rho/\rho(T=300\ K))}{dT}$ and an upper bound in the structural transition temperature.

superconductivity.

The functional form of the structural transition versus doping is unknown, however we can set bounds on the critical doping x_c. The lower bound is set by the highest doping with observable signatures of the structural transition, $x = 0.065$. Since the phase transition is concave down, a conservative upper bound is a linear extrapolation of the structural transition vs doping (Supplementary Figure 3). A linear extrapolation of the structural transition for the four underdoped compositions closest to optimal gives an upper bound of $x = 0.069$. For this manuscript we use the value $x_c^{bare} \approx x_c = 0.067 \pm 0.002$.

Finally, we note the cooperative effect from the electron-lattice coupling implies that the structural transition is always at or above the bare electronic transition temperature and, as a consequence, the bare electronic nematic quantum critical point occurs at or for lower dopings than the critical point (i.e. $x_c^{bare} \leq x_c$). In the case that our assumption of $x_c^{bare} \approx x_c$ (from the comparison of the electron and isovalently substituted $BaFe_2As_2$ phase diagrams) does not hold, x_c^{bare} can only shift to comparatively lower dopings. In this scenario, the qualitative conclusion from this work still hold. Supplementary Figure 4 shows $|m_{B_{2g}}^{B_{2g}}|$ as a function of $x - x_c$ for extremal values of x_c. Here $|m_{B_{2g}}^{B_{2g}}|$ as a function of doping still follows an apparent power law behavior, even where the temperature evolution is well described by a Curie-Weiss functional form and the extracted bare electronic transition temperature is well above zero. Quantitatively, the extracted exponent ϕ depends on x_c with best fit values of 0.9 and 1.4 for $x_c = 0.062$ and 0.05 respectively.

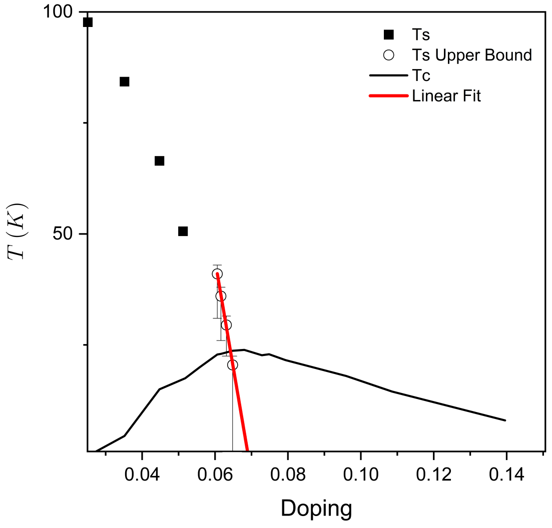

Supplementary Figure 3. **The structural transition as a function of doping.** The far underdoped structural transition temperatures (black squares) and zero field superconducting transition (black line) are from J.-H. Chu *et al.*[2] while the white circles represent the onset of the structural transition taken from the resistivity measurements at 45 T shown in Supplementary Figure 2. A linear fit (red line) of $T_s(x)$ for the four samples closest to optimal doping ($0.0606 \leq x \leq 0.0648$) puts an upper bond on the critical doping of $x = 0.069$.

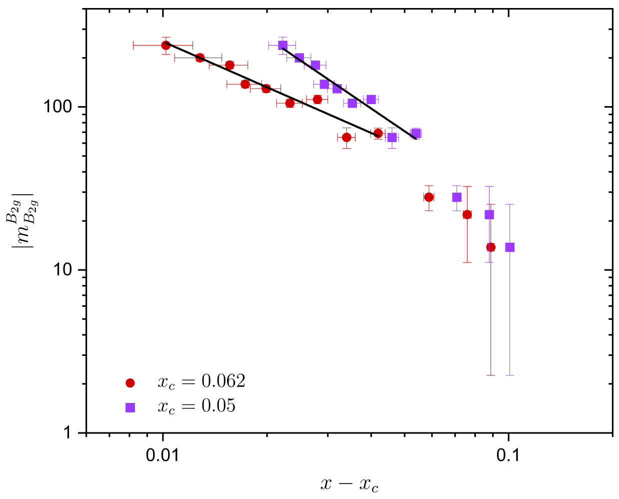

Supplementary Figure 4. **Logarithmic plot of $|m_{B_{2g}}^{B_{2g}}|$ as a function of $x - x_c$ for extremal values of x_c.** Error bars include the standard deviation of the measurement in addition to systematic errors. A fit (black line) to a power law form ($|m_{B_{2g}}^{B_{2g}}| \propto |x - x_c|^{-\phi}$) was performed over $0.0722 \leq x \leq 0.1039$ using the York computational method. The best fit values for ϕ were found to be 0.9 and 1.4 for $x_c = 0.062$ and 0.05 respectively.

C. Supplementary Note 3: Fitting ϕ

The exponent ϕ is extracted from the slope of a linear fit of $\log(|m_{B_{2g}}^{B_{2g}}|)$ versus $\log(|x - x_c|)$. The uncertainty of the fit is an approximation assuming symmetric Gaussian errors (Supplementary Figure 5) on the logarithmic scale using the York linear regression method[3] with uncorrelated x and y errors (fits performed using the "Linear Fit with X Error" analysis routine in Origin Pro 2019 and Origin Pro 2020b). The error on the logarithmic scale (σ_{log}) can be computed from the symmetric error on the linear scale (σ_x) by $\sigma_{log}^{\pm}=|log(x \pm \sigma_x) - log(x)|$, however since log is not a linear function this produces asymmetric error bars. To calculate an approximation of the standard error of our fits in this paper we linearly approximate the measurement error as $\sigma_{log} \approx \frac{dlog(x)}{dx}\sigma_x = \frac{1}{ln(10)}\frac{\sigma_x}{x}$. A comparison of these errors are shown in Supplementary Figure 5.

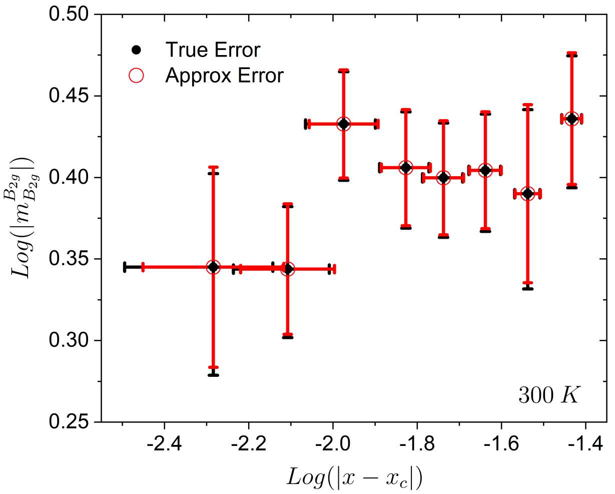

Supplementary Figure 5. **Comparison of the actual measurement error of log($|m_{B_{2g}}^{B_{2g}}(x-x_c)|$) at 300 K versus a linear first order approximation.** The symmetric error approximation (red line) is a good approximation of the absolute error (black line) in the limit of small relative error on the linear scale.

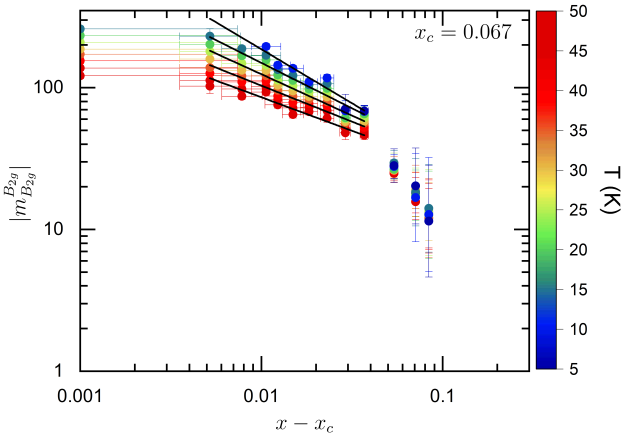

Supplementary Figure 6. **Log($|m_{B_{2g}}^{B_{2g}}(x-x_c)|$) as a function of temperature.** Data are fixed temperature doping cuts from the temperature dependence shown in Figure 1b. Data are taken in fields between 0 T - 45 T with superconductivity fully suppressed. Black lines represent power law fits. For clarity fits are only shown for data spaced every 10 K.

The fit was performed on dopings with $0.0722 \leq x \leq 0.1039$. This range was chosen since a linear fit over a rolling 5 point window is to within error constant within this range (Figure 3b). For $x > 0.1039$ the fitted ϕ begins to deviate. This is not unexpected as farther from the critical doping we expect large corrections to the scaling function. $x = 0.068$ is not included in the fit since there is large x-error due to doping uncertainty in the reduced doping $(x - x_c)$ axis. The magnitude of $m_{B_{2g}}^{B_{2g}}$ and ϕ increase with decreasing temperature (Supplementary Figure 6).

Supplementary Figure 7 shows the temperature dependence of ϕ for $x_c = 0.067 \pm 0.002$. Below 13 K, 45 T is insufficient to fully suppress superconductivity for all dopings. The ϕ value presented in the main text is taken using $x_c = 0.067$ and the error bars are the extremal error range for values of x_c between 0.065 to 0.069.

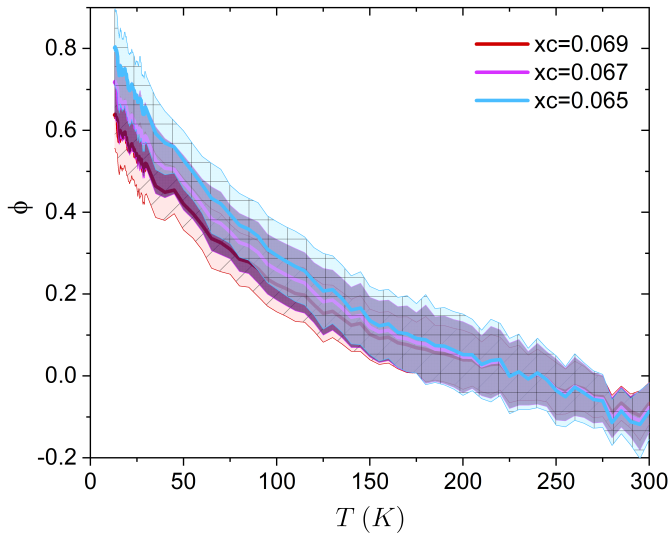

Supplementary Figure 7. **The extracted ϕ from a linear fit of log($|m_{B_{2g}}^{B_{2g}}(x-x_c)|$) vs log($|x-x_c|$) as a function of temperature for $0.068 < x \leq 0.1039$ and physically motivated values of x_c with $0.065 \leq x_c \leq 0.069$.** The fitted ϕ are displayed as solid lines and standard error for the fits are shown as shaded regions of the same color. Values for ϕ presented in the text are for $x_c = 0.067$. The quoted errors are the extremal error range from all fits with $0.065 \leq x_c \leq 0.069$.

D. Supplementary Note 4: Curie-Weiss Fits of $Ba(Fe_{0.932}Co_{0.068})_2As_2$

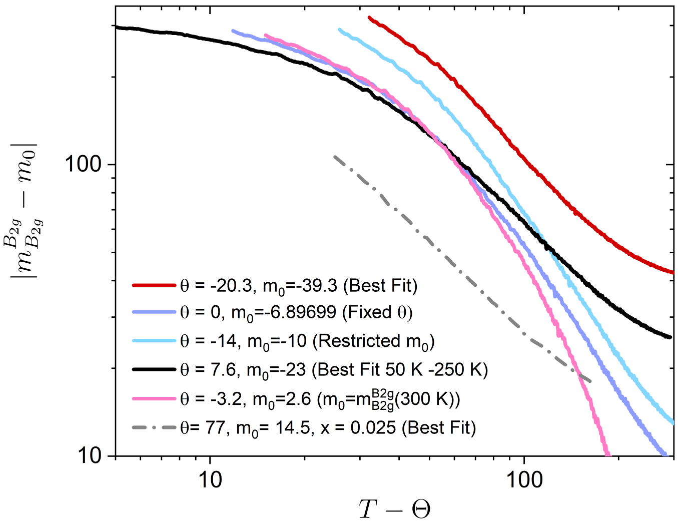

Supplementary Figure 8. **Logarithmic plot of $|m_{B_{2g}}^{B_{2g}} - m_0|$ versus $T - \Theta$ for parameters motivated from Curie-Weiss fits of $m_{B_{2g}}^{B_{2g}}$ in $Ba(Fe_{0.932}Co_{0.068})_2As_2$ (solid lines).** No physically motivated values of m_0 and Θ linearize the data over a wide temperature range. In comparison, the best Curie-Weiss fit of $Ba(Fe_{0.975}Co_{0.025})_2As_2$ from H.-H. Kuo *et al.*[1] linearizes the data of the $x = 0.025$ sample down to the structural transition at 98 K.

Prior measurements of $m_{B_{2g}}^{B_{2g}}$ for far underdoped $Ba(Fe_{1-x}Co_x)_2As_2$ have shown that it can be well fit using a Curie-Weiss temperature dependence, $m_{B_{2g}}^{B_{2g}}(\mathrm{T}) = \frac{C}{(T-\Theta)} + m_0$[1,4–6]. Here m_0 is the temperature independent background which is expected to be on the order of the geometric factor and Θ is the Weiss temperature. Near a quantum critical point Θ is expected to go through 0 K.

For near optimally doped samples a subCurie-Weiss low temperature behavior has also been previously observed[1,7]. This is consistent with our measurements. A logarithmic plot of $|m_{B_{2g}}^{B_{2g}} - m_0|$ versus $T - \Theta$ for parameters motivated

by Curie-Weiss fits for $Ba(Fe_{0.932}Co_{0.068})_2As_2$ (i.e. $x \approx x_c$) are shown in Supplementary Figure 8. These include m_0 and Θ values for the overall best fit for the whole temperature range (red line), the best fit over the whole temperature range fixing the Weiss temperature to 0 K (purple line), restricting the temperature independent term to be on the order of the geometric factor (blue line), fitting Curie-Weiss over a restricted temperature range (black line) and fixing the temperature independent value to be the room temperature value (pink line). A linear slope of 1 on a logarithmic plot would be consistent with a Curie-Weiss behavior, however no fit linearizes the data over a wide temperature window and the low temperature data diverges at a slower rate than predicted from a Curie-Weiss form for all fits. This can be compared to the Curie-Weiss fit of $Ba(Fe_{0.975}Co_{0.025})_2As_2$ from H.-H. Kuo *et al.*[1] (gray dashed line) which is linear for a wide temperature window above the structural transition at 98 K. The Curie-Weiss fit over a restricted temperature range (black line) spans the same range in reduced doping (approximately three-quarters of a decade) as the fit performed on the underdoped sample.

E. Supplementary Note 5: Goodness of Fit

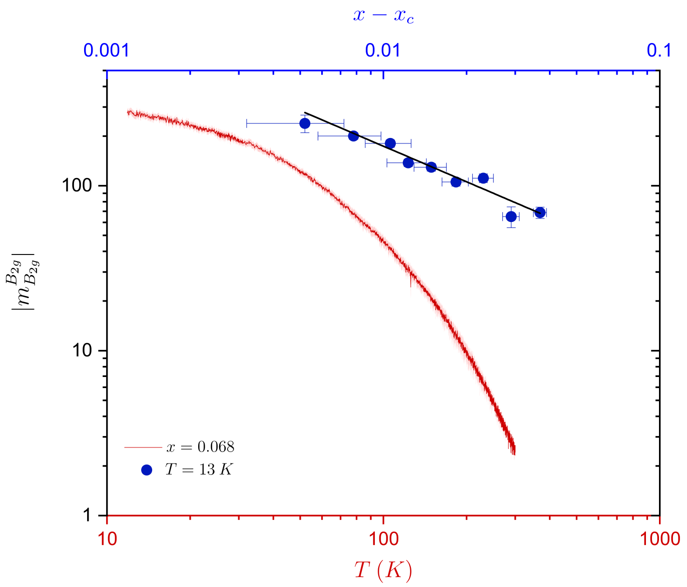

Supplementary Figure 9. **Comparison of the composition and temperature dependence of $m_{B_{2g}}^{B_{2g}}$.** The two x-axes, composition (blue, upper) and temperature (red, lower) both span two orders of magnitude. The two traces share a common y-axis. The compositional dependence is taken at 13 K and the temperature trace is taken for $x = 0.068$. The black line is a power law fit to the compositional dependence.

The compositional dependence of the elastoresistivity response is well fit by a power law functional form (Supplemental Figure 9), with a reduced chi-squared value of 0.94. This indicates that the data are well fit by this functional form and the variance is consistent with the expectation from the error. While the large errors in the composition do not constrain the fit as much as the comparatively small errors in the temperature dependence, even if we fit the temperature dependence with comparable error bars the reduced chi-squared value of a fit performed over the full temperature range is greater than one (approximately 1.9). This indicates that the behavior is not fully described by the power law functional form. Of course, for narrow temperature windows the temperature dependence can be consistent with a power law form, however the extracted exponent depend sensitively on the selection of the fit window.

F. Supplementary Note 6: Low Temperature Power Law of $m_{B_{2g}}^{B_{2g}}(T)$

$m_{B_{2g}}^{B_{2g}}(T)$ for $x \approx x_c$ does not follow a single power law as a function of temperature as shown by the nonlinear relationship of $\log(|m_{B_{2g}}^{B_{2g}}|)$ vs $\log(T)$ (Figure 4c). If the temperature dependence is converging on power law behavior

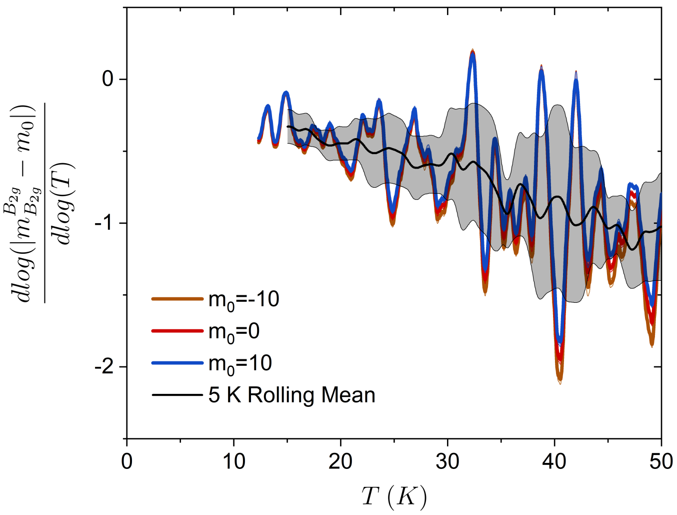

Supplementary Figure 10. **Estimation of the power law exponent of the nematic susceptibility as a function of temperature assuming the system is converging on power law behavior in temperature as $T \to 0$ K.** Colored lines are $\frac{dlog(|m_{B_{2g}}^{B_{2g}} - m_0|)}{dlog(T)}$ calculated over a 1 K window for $x = 0.068$ and $m_0 = -10$, 0, and 10. Gray line is the mean calculated over a rolling 5 K window. Error (gray shaded region), standard deviation of the mean.

as the system is tuned towards the putative quantum critical point at zero temperature then $\frac{dlog(|m_{B_{2g}}^{B_{2g}} - m_0|)}{dlog(T)}$ should approach the power law value as the system is cooled. We approximate this derivative using the slope of the linear fit of $\log(|m_{B_{2g}}^{B_{2g}} - m_0|)$ vs $\log(T)$ over a rolling 1 K window for $m_0 = -10$, 0, and 10. The result is shown in Supplementary Figure 10 along with the rolling mean over a 5 K window (gray line) and associated standard deviation of the mean (gray shaded region). The low temperature value at 15 K and standard deviation is -0.33±0.12. Overall the magnitude of the estimated power law exponent is decreasing with decreasing temperature. If this trend continues in the low temperature limit the exponent must be ≥ -0.33±0.12.

[1] Kuo, H.-H., Chu, J.-H., Palmstrom, J. C., Kivelson, S. A. & Fisher, I. R. Ubiquitous signatures of nematic quantum criticality in optimally doped Fe-based superconductors. *Science* **352**, 958–962 (2016).

[2] Chu, J. H., Analytis, J. G., Kucharczyk, C. & Fisher, I. R. Determination of the phase diagram of the electron-doped superconductor $Ba(Fe_{1-x}Co_x)_2As_2$. *Phys. Rev. B* **79**, 014506 (2009).

[3] York, D., Evensen, N. M., Martínez, M. L. & De Basabe Delgado, J. Unified equations for the slope, intercept, and standard errors of the best straight line. *Am. J. Phys.* **72**, 367–375 (2004).

[4] Chu, J. H., Kuo, H. H., Analytis, J. G. & Fisher, I. R. Divergent nematic susceptibility in an iron arsenide superconductor. *Science* **337**, 710–712 (2012).

[5] Shapiro, M. C., Hlobil, P., Hristov, A. T., Maharaj, A. V. & Fisher, I. R. Symmetry constraints on the elastoresistivity tensor. *Phys. Rev. B* **92**, 235147 (2015).

[6] Shapiro, M. C., Hristov, A. T., Palmstrom, J. C., Chu, J. H. & Fisher, I. R. Measurement of the B_{1g} and B_{2g} components of the elastoresistivity tensor for tetragonal materials via transverse resistivity configurations. *Rev. Sci. Instrum.* **87**, 063902 (2016).

[7] Straquadine, J. A. W. *et al.* Growth of nematic susceptibility in the field-induced normal state of an iron-based superconductor revealed by elastoresistivity measurements in a 65 T pulsed magnet. *Phys. Rev. B* **100**, 125147 (2019).
